# Homœopathic Therapeutics and Pathological Anatomy

**Published:** 1882-01

**Authors:** P. P. Wells

**Affiliations:** Brooklyn, N. Y.


### HOMŒOPATHIC THERAPEUTICS AND PATHOLOGICAL ANATOMY.

P. P. WELLS, M. D., BROOKLYN, N. Y.

(*Read before the American Institute of Homœopathy, 1868. Revised by the author, December, 1881.*)

Early in the controversy which arose on the announcement of the homœopathic doctrines, it was objected to them by their opponents, that both they and their author ignored the whole science of pathology, as then taught in the schools. This was cast at him as a reproach, and cited as evidence of his ignorance. It was held as proof that the doctrines he taught, were inadequate to guide the practitioner in his endeavors to apply science to the art of healing. It was thought to be conclusive as against Homœopathy, that it excluded so large a part of the science of the schools. In reply, it was shown that much of what then constituted the received science of pathology, was no better than sheer hypothesis which had but recently taken the place of other equally baseless hypotheses, and that none of these had ever been given to the profession as proved facts; that their only existence, indeed, was in the imaginations of ingenious men. In the place of these hypotheses, Hahnemann demanded a pathology based on facts. He would admit nothing till proved true, and he abstained from inculcating principles till he regarded them as abundantly established by careful and adequate observation. To replace these hypothetical doctrines he gave to the profession a philosophy of disease, which he claimed to have verified by the slow and sure process of induction; so that the reproach that he ignored the science of pathology was met by presenting to the world a better system, a true philosophy, which, being founded wholly on observed facts, he claimed should take the place of the old which was based so largely on mere imagination.

Here the two schools stood, each claiming exclusive authority for its teachings, when a new thought struck the leaders of the old. It was to study the results of diseases, as these might be visible in the changes wrought by them in the material tissues of the organism. From this has resulted the modern science of Pathological Anatomy. So the new thought has not been without its fruits. Such as these are, they are of recent growth. In the beginning of the controversy they were the property of neither school, for they had no existence. Now, when it is alleged by the old school against the new, that the latter practically rejects the science of pathology, it is to this new element of that science that reference is chiefly made. It is claimed—and not without show of reason—that this is the child of the old school; that by it this has been begotten, and has reached its present age and proportions by its own almost unaided efforts. And now, while it cherishes with peculiar love, this, its youngest darling, the sole offspring of its ripest age, and the only one which has been born of observed fact, it fails not to throw all its bitterness into the complaint that this is not received by the new school with equal tenderness, and that homœopaths do not share with the authors of this science all their fond expectations of its practical benefits.

It is forgotten apparently by the complainants that, while their care has been concentrated on this object of their love and pride, those whom they thus accuse of want of co-operation in their labors, and of appreciation of the results, have been quite as actively, and more exclusively, employed in developing and perfecting another member of the family of sciences, one of far greater importance to the art of healing than is this science of the results of diseases.

They coolly ignore the sublime labors which have given birth to a new materia medica, and presented it in its present proportions to the world. They know nothing of, and care nothing for, the toils, the watchings and waitings, from which this has grown. Nor are they more mindful of the fact that, while they of the old school have been thus busy in studying the results of diseases, the new has brought into life the pre-ordained law of healing, founded in the nature of men and things by the Power which created both. Though it had been permitted by that power that, through and by the remissness of the medical profession, it should slumber for ages with the unknown, now, just at this period of the history of science, when it began to demand demonstrated facts as its only foundation, this law was brought to light. It now lives, a power for good in the earth, solely as the fruit of the labors of the founder of the new school.

Thus it will be seen that each of the schools has had its specialties of labor and its fruit: that the old does not appreciate and accept those of the new is a loss to themselves. We do not return the reproach they cast on us, though we might with greater force; for that which they reject is more important to practical success in healing than the science which they accuse us of disregarding. It is not to be forgotten that rejecting a truth does it no harm, though many seem to think it is thereby annihilated. The skeptic is the sufferer.

When it is alleged that Homœopathy disregards the teachings of pathology, the allegation now no longer refers to that general science of disease, as at the first, but to this new creation—pathological anatomy. To this charge we are no more called to answer than members of the other school should be to that of neglecting our materia medica, or our dynamic philosophy of disease. But still we propose to reply, not for their sakes who make the charge, nor for our own, who well know how utterly baseless it is, but for the sake of some few in our own school who may be led, by the iteration of the accusation, to suppose it more plausible than it really is.

The charge is that the homœopathic school neglects and discards the teachings of pathological anatomy in their practical dealings with the sick. We begin by peremptorily denying the truth of this charge, for it is neither through a neglect, nor by a disregard of the teachings of this science, that it is not allowed a place with those elements of knowledge which control some of our practical duties. And it evinces nothing less than sheer ignorance of the proper nature and place of these teachings, as well as of the true nature of disease and its cure, to claim for them a place where, from the very nature of the case, they can never be helps, but only hindrances.

Further, to make pathology a dominant power in the selection of the curative agency, as the old school affect to do, is to entirely mistake its nature and importance, and to ascribe to it a function wholly foreign to its true relation to the practice of medicine. It is just this refusal to thus misapply the teachings of this science that constitutes the sole ground of the charge against us, which is falsely made, and which is no less than one of criminal ignorance. But it is, on the contrary, just because we are *not* ignorant, but see very clearly the true nature and relations of these teachings, that we deny them a place with those elements of knowledge which control our selection of curatives. It is a clear insight into this nature, and these relations, which manifests in the clearest possible manner that they can, from their very nature, have no place here. It does not follow from this that these teachings are without value in our estimation, or that they have no place in medical science. The exclusion of them from our process of prescription—this is the offence. Let us see how grave it is, or even if it be an offence at all against true science.

In examining this question, the first fact which arrests our attention is, that, in the act of prescribing, the two schools are not on the same ground. The old stands wholly on hypothesis, or on this as modified by a partially enlightened empiricism; while the new stands upon a no less sure foundation than an established law of nature. This difference is so great, that whatever censures the old school, from its standpoint, may cast upon the new, are, of course, of little moment. And with these censures, therefore, we have now nothing to do. We take only the one charge of excluding the science of pathological anatomy from our art of prescribing. In the outset we admit its truth, and we proceed to examine it from our own standpoint of law, that we may learn whether, in the light of this law, it is a fault or a merit.

In our office of prescribers, we are at all times bound by our allegiance to a law which we had no part in enacting, and which we cannot repeal, and dare not neglect. What it requires of us that we must do; and experience has abundantly taught us that in doing this with the utmost exactness, is our greatest practical success. Being now upon our own ground, it is not necessary to stop to prove the reality of the law—for this we assume; as also that the only known law of cure is that of similars. The question, then, before us, is simply, what does this law require of us when we attempt a cure under its guidance? Whatever this may be, when we have rendered obedience to it, we stand sinless before the world with all the bitterness and pride of its schools.

We have said the law of cure is that of *similars*. The idea of similarity involves that of comparison of two or more objects of which we can have a clear perception. The comparison which the law of cure requires is that of the elements of morbid action upon the organism on the one side, with those of the action of the curative agent on the other. On both sides, the elements to be compared must be of a character to admit of our positive knowledge of their true characteristics. Certainty in our prescriptions depends on this certainty of our knowledge; and of this certainty we are to be assured to the greatest possible degree; we must be satisfied with nothing less. If the law expressed by the formula "Like cures like," requires a comparison of the elements of the pathological anatomy of our case with similar elements (viz: the ascertained results of the action of curative agent), we are, no doubt, to yield obedience to the demand. If not, then only to the law are we responsible, and not at all to the cavils or complaints of those who reject the law. But the law does not require this comparison. This is evident from several considerations. The first is, that there is, in most cases of acute disease, an initiatory stage, in which they are curable to a far greater extent, and with greater certainty and ease, than when they have passed beyond it. It is in this stage that there is the greatest amenability to curative agencies. There is, as yet, no change wrought in the material tissues of the organs: consequently there is no pathological anatomy, and none of its elements can therefore be obtained for the comparison required by the law. If these elements are necessary to a sure and successful prescription, then here, at this point in the history of cases, where they are most curable, they should be found in the greatest numbers. And yet it is just here that they are not found at all. This is perfectly conclusive as to any necessity there may be for the consideration of the facts of pathological anatomy in determining the choice of the curative agent. We may go farther, and declare plainly that the law of cure never demands any comparison in which these facts are of necessity to be embraced. If it were otherwise, then the law would require that this large class of curable cases (the more curable, the nearer the prescription approximates to the initiation of the attack), be allowed to pass on without prescription, till diseased action has developed the required pathological changes in the tissues of the organs, before presuming to attempt a choice of a remedy for the case. There can be no greater absurdity than to suppose this to be one of the conditions of a divinely appointed law, the object of which, is to secure certainty and success in the cure of the sick.

There is another objection to our acceptance of the elements of this science into the group of facts, which are the proper objects of comparison, when prescribing according to the law of cure. We have seen that *certain* knowledge, as to the true nature of the facts to be compared, is indispensable to their being admitted to our consideration in the discharge of this duty. The fact that *certainty* as to the pathological conditions is only attained by inspection after death and dissection, is fatal to their claim to be received into the company of those other elements of the case, which may be fully examined and understood during life, and which alone must determine the choice of the curative remedy. It is not to be denied that the pathological anatomy of any case, even after it has so far advanced as to develop changes in the material tissues of organs, is, during life, to a considerable extent, a matter of conjecture. The best that can be said of it is that the conjecture is probable. This is to fall far short of that certainty which the law of cure demands. It is reasonable to insist that knowledge obtained only after death, comes altogether too late to be of use in finding the cure for the living sick.

We have still another objection to the claim set up for this class of facts. It is this: The relation which the divinely appointed law has established between the diseased condition and that external agent which by the law is its natural cure does not exist, in any degree, on the part of the disease, in those elements of its condition which constitute its pathological anatomy, but altogether in other elements of the case, easy of recognition, and admitting of certainty of knowledge of their true character, so far as this relationship of law is concerned. The truth of this assertion is abundantly sustained by the practical successes of those who have made this last class of facts, the basis of their decisions in prescribing for the sick, to the exclusion of the other class urged upon them by those only who were ignorant of the spirit of the law which has ever proved itself a safe and sufficient guide. The successes of Hahnemann, Gross, Stapf and Bœnninghausen, and of others like them in Europe; and of Hering, Haynel and Dunham, with many others in our own country, have been so obtained. Not that these masters were ignorant of the science they have been accused of neglecting; but they saw clearly its uses, and restricted themselves to these, instead of attempting to derive from it information which it never did and never can impart.

On the other hand, the truth of this assertion is equally sustained by the failure which has attended the endeavors of those who have sought to incorporate the facts of this science into a practice otherwise founded, in part at least, on the homœopathic lww. Only disappointment has followed this course, as, from the nature of the case, it only could. The more and the more frequently they were unsuccessful, the harder have they striven to make a more exact application of what they regarded as the science, *par excellence*, of their calling, viz: that of pathological anatomy—to the demands of their cases, till disappointment, often repeated, has begotten scepticism, and scepticism has ended in apostasy, and apostasy in disgrace.

It was, however, due only to the incompetency of the individuals who were thus trying to improve the law of nature by adding to it that of which it had no need, and which it would not accept. The practitioners who have relied upon this science in their prescriptions have not been the successful men of our school.

There is another objection to the use of the teachings of this science as urged upon us by our opponents. It is this: the only knowledge we can have of these facts, during the life of the patient, is derived from the *symptoms* of the case. These are just as available to the prescriber, without attempts to refer them to *supposed* changes in the tissues of internal organs as with; indeed, they are much more so. Taken of themselves, they form a part of the living facts with which we have to do. But, taken as exponents of changes which may or may not have been wrought in the tissues by the morbid process with which we are dealing, they are always extremely uncertain, and not unfrequently, wholly deceptive. It is well known, and post-mortem dissections have abundantly established the fact, that symptoms so similar as to admit of no distinction, have been connected with pathological conditions which were even the exact opposites. This has been well shown by that eminent philosopher and physician, Abercrombie, in his treatise on diseases of the brain. The same has been observed by many others, who, like Abercrombie, have been rather puzzled than instructed by it. In such cases, as we *cannot* know to which of the opposite states we are to refer the symptoms, we have no resource but to guess. If the greatest possible certainty, and not conjecture, is to be sought and secured in our prescriptions, then this attempt to incorporate into the basis for them an element so largely made up of hypothesis, must be abandoned. To this we may the more readily assent, as it has hitherto contributed nothing to practical success, and only needlessly and uselessly complicated the process by which alone it can be attained. The above objections to this use of pathological anatomy are wholly from its failure truly to indicate the disease. It will be readily seen, from what has been said, that there must be a similar failure to indicate the drug. A prescription, according to the law of cure, is the finding of a drug which has the power to produce, and which has been known to have produced, certain effects on the healthy organism, like to the phenomena of the disease to be treated. These are the phenomena by which the disease is related to the drug as its curative. If the facts of the science we are considering belong to that class, then we are to find, in the known effects of the drug changes in the material tissue of organs, the result of its action, similar to those which are developed by the disease, the result, on the other hand, of the morbid action. In order to attain this knowledge of the changes which drugs can and will effect on the tissues of organs, it will be necessary to push the proving on the healthy to an extent of poisoning, and the poisoning to the extent of destructive changes in the organs of the body, and beyond this, even to the destruction of life, before the required knowledge can be obtained. For, as on the side of the disease, the facts of this science have only been gathered by dissection, so, on that of the drug, if the law of cure requires similar facts, the result of drug-action, to be brought into comparison with those which dissection has disclosed as the result of disease, they can only be obtained by the same process. Every prover of the drug must be dissected, and each patient must share the same fate before we can certainly know that the changes wrought by the two agencies are similar. And, as drugs do not affect all persons alike, the sacrifice of one prover for each drug, and one patient for each disease, would be wholly insufficient. This certainly presents the claim and the charge, so often and so ostentatiously paraded by the old school, in a sufficiently absurd light. And it is possible, that the stolidity which has failed or refusud to see the superiority of the results of a practice founded on law, over those of a practice founded on hypothesis, might see that prescriptions in such a case would be of no great value. But the difficulty of introducing the facts of this science into the process of prescribing, under the guidance of the law of similars, is further increased by the fact that the changes of tissue which constitute them, are from day to day themselves changing; so that the exact conditions of the pathological anatomy of a case known yesterday, have ceased to be the exact conditions of the same case when we visit it to-day. And this is repeated from day to day as the case proceeds. This would necessitate daily examinations, such as we have shown to be requisite in the first instance, as long as the duty of prescribing for the case should continue. It may be objected to this view of the case, that it contemplates prescribing under the guidance of one law only—that of similars. This objection can come only from the side of the old school. The scope of this paper attempts no reply to them. Its aim is to show the less instructed and younger members of our own school, the absurdity of the claim set up for this misuse of pathological anatomy. For their sake we reply; and we do so by asking the objector if by this he means to intimate that prescribing with the unlimited license of hypothesis is better than that under the guidance of law? If not, then we would further ask if he knows of any other law than that of similars, available for the guidance of the prescriber? If so, then we ask, first, for the evidence of its existence even; and secondly, for the evidence of its superiority to the law given us by Hahnemann, which we have proved and accepted. And we may here say that we can accept no other evidence of this superiority than well-attested clinical results, superior to those which have followed practice based on the law of similars. To such evidence we are all most assuredly bound to yield, and to give to a law so attested the same hearty allegiance we render to that which we now know and trust. In the absence of all evidence that any such other law exists, and in view of the abounding evidence of the superior results of a practice founded on our law over that based on either hypothesis or empiricism, we claim to be fully justified in making this success a full answer to the claims of pathological anatomy as a guide in prescription.

We appeal to this, the only known law of cure, and, in its light, show the utter absurdity of the claim. This is our defense against the charge of ignorance, because of our refusal to so admit them; and this is the defense of homœopathy when it is charged with inadequacy as a guiding law in the treatment of the sick, because it excludes, not helps, but hypotheses, which are hindrances.

So far, we have argued this matter as related to our school, and wholly in the defensive, as against those who have sought, by this absurd demand and charge, to discredit an entire school and practice. It would have been well, before they made this futile attempt to disparage, that they had been better acquainted with its principles. It is easy to believe, that, had they known more, they would have spoken less. We now leave the defensive as to this charge, and assert that the attempt to subject their prescriptions to the control of the facts of this new science has been an utter failure on the part of the old school in their treatment of the sick without law. The supposed facts are as utterly useless in a practice which recognizes no law of cure, as in one guided by the law of similars; and, to sustain the truth of this, we appeal to the records of such practice, and particularly to those of the results of the treatment of that disease best studied in its pathological anatomy, Typhoid Fever. It may be unhesitatingly affirmed of this fever, and of its treatment without law, that it is no more successful to-day than when Louis made his first dissection in the series which settled the pathological anatomy of the disease. Louis himself never pretended that his discoveries had modified his own prescriptions. It is interesting to follow him in this series of laborious studies, and see how his prescriptions in succeeding cases are not in the least affected by the knowledge he had gained of the localization of the morbid process and peculiar changes in the tissues invaded. They were the same in the last of the series as in the first. He never pretended it was otherwise. Nor did he pretend that they threw such light on the relationship of the fever to its curative agencies as should induce any other man to modify his prescriptions for typhoid fever. If the facts discovered by M. Louis have so modified the hypothetical or empirical treatment of this fever by any man as that he has thereby obtained a better success than attended the practice of intelligent men before these discoveries, we have failed to learn the fact. The most that has been claimed for them, a modifiers of practice, is rather on the score of their having discarded remedies than of having suggested any; that by them physicians have been induced to give fewer and less irritating drugs—in plain English, that they have been instructed to do less mischief than before, not to do more good. And is this all? And has the cry from those who have learned so little been so loud, and sometimes even so offensive, because others, who have needed no such restraints, have refused to encumber their better practice by an accessory which has brought to themselves no greater good? We go one step farther; we assert that the claim that the facts of this science have beneficially modified the practice of hypothesis or empiricism in other diseases, to a greater extent than in this fever, is false. They have suggested no useful remedies to the old school itself; but it has been reproaching the new, because it refused to them authority to modify its practice founded on law. It is a sufficient reply to this impudent claim to be our teachers in the matter, as set up by these gentlemen, *first cast the beam out of thine own eye.* We say to the gentlemen of the old school, show a better clinical record than your predecessors, and prove its superiority to be owing to the aids you have derived from pathological anatomy, before you attempt to discredit your compeers for rejecting it from theirs. Go farther, and show a better record, with this new aid than the new school shows, having rejected it, and you have won the victory in the controversy. By any other course, you expose yourselves to the suspicion that you are already conscious of the weakness of the position you occupy, and of the charge you have brought. Show that you have fully and fairly admitted these facts to the control of your own prescriptions, and that the result is a greater success than attends our practice founded on law, and you will merit respectful attention. Unless you do this, your present course suggests a resemblance to the policy of that animal who would have his neighbors mutilated to escape the singularity of his own misfortune. The suspicion will be that you have in your own practice found the resort a failure. With this hint we take leave of these complainants.

The laws of nature are the appointments of that Supreme Being, who, in their ordination, comprehended all their relations and results from the beginning. In their enactment he could by no possibility make a mistake. One of these laws declares the curative relationship between diseases and certain drugs. Like all the rest, this law exists, not in the imagination or edict of any man or school, but in the very constitution of men and things, as ordered in the beginning by this Almighty Power. The intelligent world glories in the fact that all natural laws have been ordained in infinite benevolence, as well as in infinite wisdom and power. Is this natural law of curative relationship an exception, and the only one in all the domain of God's natural government? (Where is the evidence of the fact, and if so, why?) If this relationship at all embraces the facts of the science we have been discussing, and calls for a consideration of them in the curative prescription, then we submit that we have already sufficiently shown that this law is altogether a mistake. The benevolence so conspicuous in all the rest is wholly wanting in this, because its application is an impossibility. The law in this case, is simply a trifling with human necessities. The Author of all good has never so mocked his dependent creatures. He has not included the facts of this new science in the elements of disease and drug-action, in the similarity of which He has ordained the law of cure. A clear appreciation of these elements will be quite sufficient to show that the exclusion of these facts from the scope of the law is of Divine appointment, and that we, of the new school, have only recognized this arrangement, and are in no way responsible for its existence. It is certainly most clear that, if a God of infinite wisdom and benevolence had determined a law of cure for the relief of the suffering sick among mortals, and had really purposed that these should be healed by virtue of a compliance with its provisions, both these attributes of the Divine Being would have combined to establish the law in ascertainable relations, between diseases and the agents ordained for their cure, such as should be most obvious to the senses, and most readily available for the purpose for which the law was ordained; and more, that these relations would assuredly be such as could be known and understood, and by no means such as must of necessity be attended with more or less doubt and uncertainty during life, and least of all in any which must rest largely on conjecture, or only to be known after death. This is precisely the arrangement which has been made in the enactment of the law of cure by similars, in accordance with these Divine attributes. Its relationships are wholly in those elements of the two classes of objects of comparison, the easiest of perception and comprehension, so far as is necessray to a compliance with the demands of this law. This has been happily secured in the selection of the elements in which these relationships are fixed; for a selection of these there certainly is, and such a selection as would hardly have been made by any other than the Author of this law. At the first glance, it would seem to have been best to select these elements always present in any form of disease, and make these the basis of comparison on the one side, and then there would be only the small difficulty of finding a single drug, which in its action on the living organism presents similar elements; and the whole difficulty of prescribing for this disease, for all time and in all cases, would be at an end. This is precisely what the Founder of this law has not done. He has given to it a much broader scope, and by this has relieved it of an uncertainty which must otherwise have inhered in it forever. If the law had been fixed in those elements which characterize the class, then embarrassment would have arisen from the number of agents capable of presenting, in their action, phenomena similar to this class, as present in the case to be treated; and the only resort would be to give them in succession, till we should hit upon the right one. This would cause great delay and danger. The Maker of the law did not so ordain.

As illustrative of this, suppose the case to be treated one of simple dysentery. It has frequent, small discharges from the rectum, of mucus or blood, or both, accompanied with pain, tenesmus and fever. This group of symptoms is present in all cases of the disease. On turning to the materia medica for a remedy, the prescriber is met by more than a score of drugs which have produced this group of symptoms on the healthy, living subject. What will he do? If he is a beginner, he will do as all of us have done, till we learned better—give one of the number and if it does not succeed, another, and so on. He will only be realizing the disappointments we have all encountered, till we found that the similarity which the law requires is not in these generic elements of either the disease or the drug.

But the chief difficulty, and that which made the selection of generic groups as the domain of the law impossible, is the fact that they do not constitute the whole case; and also, when they are found in any succeeding case, they are by no means a repetition of identities. Though there is a general resemblance of common features in this generic group, it is, in each case, surrounded by, and associated with, concomitants, which declare the specific nature of each case in this group as it occurs. By these the truth is made clear that the resemblance of the generic phenomena of cases is superficial and apparent only, and not real. These concomitants stand in each case as the exponents of its true character, and they alone declare what *kind* of case it is with which we have to do. The generic phenomena proclaim the family to which the case belongs; the concomitants of these, or the specific phenomena, the particular member of the family which we have just met. It is the resemblance to the features of this particular member of the family, which the law has made it incumbent on us, in our office as prescribers, to find in the effects of some one curative agent on the living organism; because it is just in these specific features of disease that Infinite Wisdom and Benevolence has established the domain of the law of cure, so far as the disease itself is concerned.

On the side of the drug there was, of necessity, a similar selection of elements from the sum of the effects produced on the organism by particular drugs and for a similar reason. The generic, or those common to several drugs, were excluded; and those which belong to the particular drug, and characterize it, were selected. And these on the one side, and those which declare the specific character of the disease on the other, were the precise elements of the two objects of the comparison in which Almighty Power and Wisdom placed the law of cure. Resemblance of these is the *like* which cures. The law of the similars requires and accepts no other similarity. Hence the facts of the science of pathological anatomy have been excluded from the requirements of the law by its Great Author in its original constitution; and he who attempts to thrust them in, where they have been thus excluded, is likely to have no great success. It follows, also, that their rejection from the prescriptions of our school, so far from being any sin against science, is only a compliance with the requirements of the great controlling natural law. But it is often and plausibly asserted that it is certainly our duty to study the nature of disease as disclosed by its results, meaning by this the facts of pathological anatomy. To this there can be no objection, and we do not object when we assert that the light which these facts throw on the nature of disease does not reach at all to its curative relations, as we have shown, but is limited solely to that power to produce in the tissues the changes from which these facts have had their origin. The nature of disease, thus far, is disclosed by these facts, and here the revelation stops. There is a seeming of scientific wisdom in the assertion of this duty, calculated to mislead the beginner as to the extent of the benefits to be derived from the investigation recommended. If, in its results, he expects, as many seem to have done, a disclosure of the whole nature of disease, he is only to be disappointed. He will simply learn that it is the nature of this or that disease to effect this or that change in the anatomical constitution of the tissues of certain of the organs of the body. Beyond this he can learn of its nature absolutely nothing at all. We have denied that we undervalue the knowledge which this science reveals. We add that it is no contradiction of this denial when we limit its application, as above set forth. We deny that the limitation is ours. It is in the nature of the facts of the science itself, and in that of the general nature of disease, and of the law by which God ordained it should be cured. We simply recognize the fact, and receive it as it has been given into our hands by the Power which has established this limitation, with the other facts of nature. If this power has limited the proper application of the knowledge of this science to that part of our practical duties which deals with the diagnosis and prognosis of diseases, we have no power to change the arrangement, and we do not see how any benefit could be educed from it if we had. It having been so limited and connected, there can be no wisdom in endeavors to force on this branch of knowledge functions which can never belong to it, or in anticipating any light on other elements of the nature of disease which it can never shed. It is the part of true wisdom here, as in other departments of natural science, to discover the divinely appointed arrangement, to receive it, act upon it, and therewith to be content.

